# Kinetic Study of *Coprinus cinereus* Peroxidase-Catalyzed Oxidation of 2,2′-Dihydroxyazobenzene

**DOI:** 10.3390/ijms25020828

**Published:** 2024-01-09

**Authors:** Rūta Ivanec-Goranina

**Affiliations:** Department of Chemistry and Bioengineering, Faculty of Fundamental Sciences, Vilnius Gediminas Technical University, 10223 Vilnius, Lithuania; ruta.ivanec-goranina@vilniustech.lt; Tel.: +37-065-138-643

**Keywords:** azo dyes, environmental pollutants, 2,2′-dihydroxyazobenzene, peroxidase, biocatalytic oxidation

## Abstract

Azo dyes are of concern due to their harmful effects on the environment and human health. The oxidation of 2,2′-dihydroxyazobenzene (DHAB) catalyzed with recombinant *Coprinus cinereus* (rCiP) peroxidase was investigated. The kinetic measurements were performed using the spectrophotometric and fluorimetric methods. The dependences of the initial reaction rates on enzyme, substrate and hydrogen peroxide concentrations during DHAB oxidation were established, and bimolecular constants of enzyme interaction with DHAB were calculated. This research demonstrated that the initial biocatalytic oxidation rates of DHAB depend on the pH and the estimated pKa values of the active forms of rCip. This study’s findings thus contribute to a more comprehensive understanding of the biocatalytic oxidation of DHAB, providing valuable data for assessing the long-term toxicity, carcinogenesis and epigenetic effects of azo dyes in the environment.

## 1. Introduction

**Figure 1:**
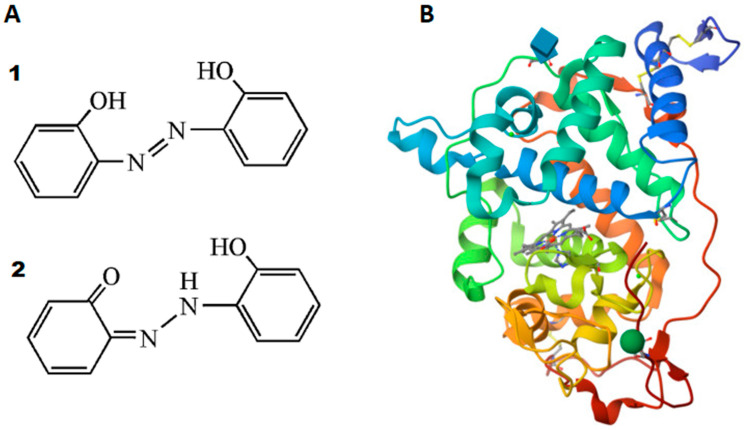
(**A**) Two tautomeric forms of DHAB: (**1**) azo form and (**2**) hydrazone form [13]. (**B**) Structure of rCiP determined to be 2.0 A by X-ray diffraction method (the different colors indicate the alpha-helices that surround the heme group (light purple) inside) [29].

Azo dyes, characterized by the presence of the azo (-N=N-) chromophore, represent a prominent class of organic compounds widely employed in various industries, ranging from textiles to biomedical applications [1,2,3,4]. The distinct physical and biological properties of azo dyes have garnered significant attention, propelling research endeavors to elucidate their behavior in both synthetic and biological contexts. Azo dyes are renowned for their vivid and diverse color palette, making them pivotal in the textile and food industries. The ability to modulate their chemical structure allows for the tailoring of colors, a property extensively harnessed in the production of colored fabrics and consumer goods [5]. Recent studies have delved into the photophysical properties of azo dyes, revealing intriguing phenomena such as photoisomerization. This property is instrumental in the development of photo-responsive materials, including liquid crystal displays and light-sensitive polymers, showcasing the versatility of azo compounds in material science [5,6,7,8]. Effects such as photo-switchable and antiproliferative effects of azo compounds have also been actively investigated [9,10]. Azo dyes have demonstrated biocompatibility, prompting exploration in drug delivery systems. Recent research highlights their potential as carriers for targeted drug release, emphasizing their role in advancing biomedical applications [4,11,12]. One such azo dye is the well known 2,2′-dihydroxyazobenzene (DHAB), which has two tautomeric forms: the azo form and the hydrazone form (Figure 1A). DHAB is a highly fluorescent molecule and can coordinate very well with many metal cations, so it is applicable in the detection of various metal ions [13]. Photoactive polyurethanes based on fluorescent DHAB segments were also synthesized [14]. For these aforementioned reasons DHAB was chosen in this study.

Due to the wide range of applications of azo compounds, the safe disposal of azo dyes is especially important. Unfortunately, the presence of these dyes in the aquatic ecosystem is the cause of serious environmental and health problems related to toxicity and carcinogenicity [15,16]. Different methods can be used for the degradation and decolorization of azo dyes, but the enzymatic method has so far attracted increasing interest for the purification and/or degradation of azo dyes from wastewater as an alternative strategy to conventional physicochemical treatments [17]. Enzymatic methods for azo dye degradation and decolorization have great potential due to their environmental friendliness and cost [15]. White rot fungi can degrade many complex compounds due to the extracellular enzymes they produce. Most of them are oxidases and peroxidases with very high oxidative capacity. These enzymes can oxidize the dye structures to form lower molecular weight and often (but not always) less toxic compounds [18]. One such enzyme is fungal *Coprinus cinereus* peroxidase (CiP; identical to *Arthromyces ramosus* peroxidase), which has attracted considerable attention due to its high specific activity and wide substrate specificity [19]. Studies have demonstrated the effectiveness of CiP in degrading azo dyes through enzymatic processes. The focus has been on optimizing the reaction conditions, including pH, temperature and enzyme concentration, revealing the factors that influence the degradation kinetics. The effect on the color and structural properties of azo dyes during rCiP degradation was also investigated [20,21,22,23]. Developing genetic engineering technologies creates new opportunities to synthesize enzymes by gene recombination. Recombinant gene products (enzymes) are more readily available and as effective as their non-recombinant counterparts [24,25]. Thus, recombinant CiP (rCiP, Figure 1B) was constructed by genetic engineering methods and expressed in an *Aspergillus oryzae* strain. Nuclear magnetic resonance, protein chemistry and Raman scattering spectral analyses revealed that rCip is identical to CiP [19,26], and it even showed higher stability compared to CiP [27,28]. For these reasons, rCiP was well suited for this study.

Peroxidases incur a cyclic reaction when they react with azo dyes. This sequence is summed up in the reactions below [19]:

(1) $$E+H_{2}O_{2}\overset{k_{1}}{\to }\mathrm{cpdI}+H_{2}O$$

(2) $$\mathrm{cpdI}+\mathrm{DHAB}\overset{k_{2}}{\to }\mathrm{cpdII}+{\mathrm{DHAB}}^{•}$$

(3) $$\mathrm{cpdII}+\mathrm{DHAB}\overset{k_{3}}{\to }E+{\mathrm{DHAB}}^{•}+H_{2}O$$

According to this classical ping-pong scheme, the active site of the enzyme (E) interacts with hydrogen peroxide (H_2_O_2_, which reduces it in a two-electron manner), followed by a transformation of the active site (Equation (1)). The modified enzyme (cpdI/cpdII) oxidizes DHAB to a free radical (DHAB^•^) in a one-electron way in two stages (Equations (2) and (3)). k_1_, k_2_, k_3_ are the reaction rate constants of Equations (1)–(3).

To the best of our knowledge, rCiP has not been applied to the oxidation of the previously mentioned azo dye 2,2′-dihydroxyazobenzene (DHAB) until now. This study focused on the kinetics of DHAB oxidation using rCiP. The main objective of this study was to determine the kinetic parameters of rCiP-catalyzed DHAB oxidation.

## 2. Results and Discussion

### 2.1. Dependence of the Initial Biocatalytic Reaction Rate on pH 

**Figure 2:**
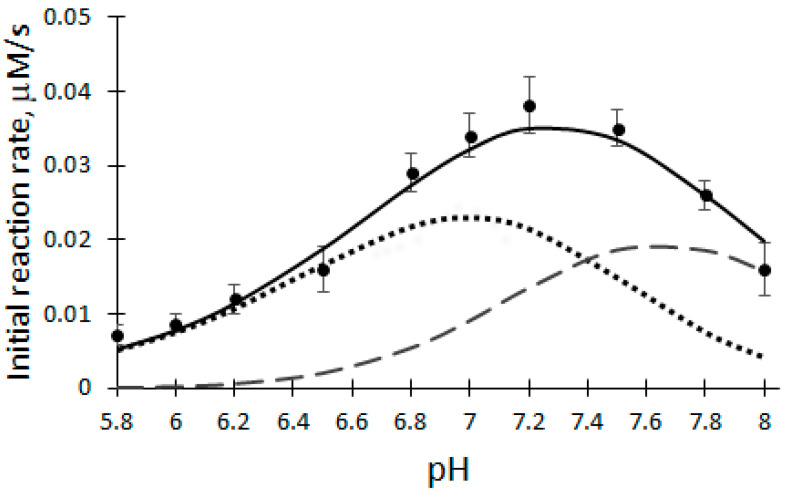
Dependence of the initial reaction rate on the pH of the buffer solution catalyzed by rCip. Dashed lines—active forms of enzyme F1 and F2; solid line—deconvolution curve.

The pH dependence of the initial reaction rate (V_0_) of the biocatalytic oxidation of DHAB was investigated at a temperature of 25 °C, 100 μM H_2_O_2_, 12 μM DHAB and 5 nM rCip, in a 50 mM phosphate buffer solution of pH 5.8–8.0 (Figure 2).

In order to explain the obtained results, mathematical modeling and optimization of the initial reaction rate (V_0_) dependence of DHAB biocatalytic oxidation on pH values were performed at steady-state conditions. The pH dependence of the initial reaction rate of rCip catalyzed by DHAB oxidation was analyzed using three proton transfer schemes [30]:

(4) $$\begin{array}{c} \;\;K_{1}\;\;\;\;{\;K}_{2}\;\;\;{\;K}_{3}\\ F3↔F2+H^{+}↔F1+H^{+}↔F+H^{+} \end{array}$$

where K_1_, K_2_, K_3_ are the dissociation constants of singly, doubly and triply protonated forms of cpdI (cpdII), respectively, and F + H^+^ is the native form of rCip. 

According to Equation (4), when the F1 and F2 forms are active (Figure 2, dashed lines), the pH dependence of the initial reaction rate (V_0_) of DHAB biocatalytic oxidation is described by the following equation [30]:

(5) $$V_{0}=V_{\max}\cdot \left(\frac{\frac{\left[H^{+}\right]}{K_{3}}}{\left[1+\frac{{[H}^{+}]}{K_{3}}+\frac{{{[H}^{+}]}^{2}}{K_{2}K_{3}}+\frac{{{[H}^{+}]}^{3}}{K_{1}K_{2}K_{3}}\right]}+\frac{\frac{{{[H}^{+}]}^{2}}{K_{2}K_{3}}}{\left[1+\frac{{[H}^{+}]}{K_{3}}+\frac{{{[H}^{+}]}^{2}}{K_{2}K_{3}}+\frac{{{[H}^{+}]}^{3}}{K_{1}K_{2}K_{3}}\right]}\right)$$

Therefore DHAB oxidation was modeled in the pH range of 5.8–8.0 at steady-state conditions using Equation (5). Because it was established that V_0_ of biocatalytic DHAB oxidation depends on the pH of the buffer solution, the optimal pH for further experiments was chosen to be 7.2. Taking into account the number of experimental points, the obtained correspondence of the approximation is high (the coefficient of variation obtained is equal to 10.8%). pKa values of rCip active forms: 6.7; 7.4; 7,8.

### 2.2. Dependence of the Initial Biocatalytic Reaction Rate on H_2_O_2_

**Figure 3:**
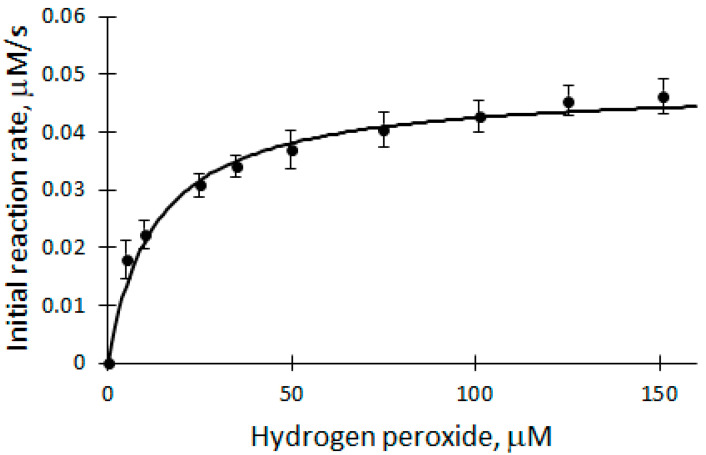
Dependence of the initial reaction rate of biocatalytic DHAB oxidation on H_2_O_2_ concentration: 12 μM DHAB, 5 nM rCip, 5–150 μM H_2_O_2_.

It is known from the literature that excessive concentration of H_2_O_2_ inactivates the enzyme. Therefore, a study of the H_2_O_2_ dependence of the initial reaction rate was carried out to find out whether the concentration of H_2_O_2_ is suitable for further analysis of biocatalytic DHAB oxidation. The dependence of V_0_ on the concentration of H_2_O_2_ was studied in the range of 5 to 150 μM (Figure 3) at a temperature of 25 °C in a 50 mM phosphate buffer solution of pH 7.2.

The ongoing process is described using the Michaelis–Menten equation. The apparent kinetic parameters V_max_ = (4.8 ± 0.2) × 10^−8^ M/s and K_m_ = (1.3 ± 0.7) × 10^−5^ M/s were obtained. From the graph (Figure 3), it can be seen that 100 μM H_2_O_2_ used in the experiments is at the saturation level, which means that the concentration of H_2_O_2_ does not limit the biocatalytic oxidation reactions of DHAB (Equations (2) and (3)) and is suitable for analysis.

### 2.3. Dependence of the Initial Biocatalytic Reaction Rate on rCiP 

**Figure 4:**
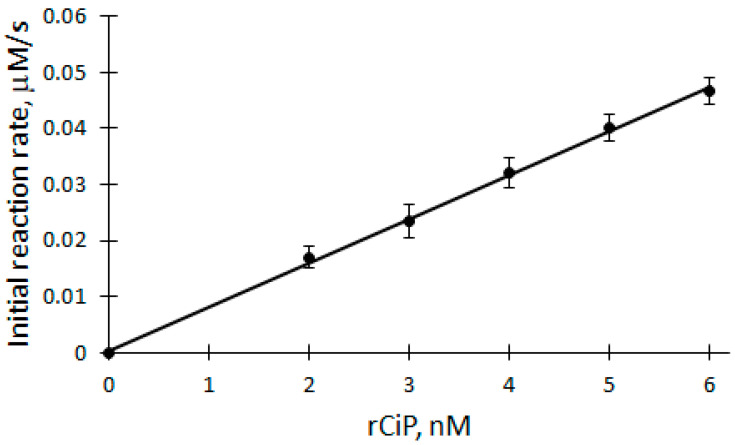
Dependence of the initial reaction rate of DHAB biocatalytic oxidation on rCip concentration: 100 μM H_2_O_2_, 12 μM DHAB, 2–6 nM rCip.

The dependence of biocatalytic oxidation V_0_ on the concentration of rCip was studied in the range of 2 to 6 nM at a temperature of 25 °C in a 50 mM phosphate buffer solution of pH 7.2 (Figure 4). A linear equation was used to analyze the rCip dependence of V_0_ of the biocatalytic oxidation of DHAB (correlation coefficient r = 0.9992). The apparent bimolecular constant k_ox_ is calculated according to the following formula:

(6) $$k_{\mathrm{ox}}=\frac{\mathrm{slope}\left({\left[E\right]}_{t},V_{0}\right)}{2[\mathrm{DHAB}]}$$

where slope is the tangent of the angle of inclination of the line (tg(α)).

Applying Equation (6) and based on the parameters obtained from the dependence, the apparent bimolecular reactivity constant of DHAB with rCip equal to 3.46 × 10^5^ M^−1^s^−1^ was calculated. 

To explain the results obtained, mathematical modeling of steady-state kinetics was performed. According to the classic scheme of action of ping-pong peroxidase (Equations (1)–(3)), to derive the initial reaction rate, a system of differential equations expressed in terms of time variation of the concentrations of the enzyme (E) and enzyme complex I (cpdI) participating in the reaction was solved:

(7) $$\left\{\begin{array}{c} \frac{d\left[E\right]}{\mathrm{dt}}=k_{3}\left[\mathrm{cpdII}\right]\left[\mathrm{DHAB}\right]-k_{1}\left[E\right]\left[H_{2}O_{2}\right]=0\;\\ \frac{d\left[\mathrm{cpdI}\right]}{\mathrm{dt}}=k_{1}\left[E\right]\left[H_{2}O_{2}\right]-k_{2}\left[\mathrm{cpdI}\right]\left[\mathrm{DHAB}\right]=0\; \end{array}\right.$$

where t is the time, and [E], [DHAB], [cpdI], [cpdII] and [H_2_O_2_] are the concentrations of the components involved in the reactions, respectively. Equation (7) are set to zero, since stationary kinetic conditions were assumed in the simulation. The material balance equations of the enzyme were also written down:

(8) $$E_{t}=E+\mathrm{cpdI}+\mathrm{cpdII}$$

where E_t_ is the initial enzyme concentration. After mathematical steady-state kinetic modeling operations, the initial rate of DHAB oxidation is obtained as follows [31]:

(9) $$V_{0}=\frac{2k_{1}k_{2}k_{3}{\left[E\right]}_{t}\left[\mathrm{DHAB}\right]\left[H_{2}O_{2}\right]}{k_{2}k_{3}\left[\mathrm{DHAB}\right]+k_{1}k_{3}\left[H_{2}O_{2}\right]+k_{1}k_{2}\left[H_{2}O_{2}\right]}$$

where [E]_t_, [DHAB] and [H_2_O_2_] are the concentrations of the enzyme, 2,2′-dihydroxyazobenzene and hydrogen peroxide. After simplifying the expression of Equation (9), where the rate-limiting constant (k_ox_) describes the slower rate of the cpdI or cpdII reaction (if k_3_ > k_2_, then k_ox_ = k_2_; if k_2_ > k_3_, then k_ox_ = k_3_), the following expression can be used:

(10) $$V_{0}=\frac{2k_{1}k_{\mathrm{ox}}{\left[E\right]}_{t}\left[\mathrm{DHAB}\right]\left[H_{2}O_{2}\right]}{k_{\mathrm{ox}}\left[\mathrm{DHAB}\right]+k_{1}\left[H_{2}O_{2}\right]}$$

Also, applying Equation (10), where k_1_ = 7.1 × 10^6^ M^−1^s^−1^ [32], the apparent bimolecular reactivity constant of DHAB with rCip was calculated to be 3.31 × 10^5^ M^−1^s^−1^.

### 2.4. Dependence of the Initial Biocatalytic Reaction Rate on DHAB

**Figure 5:**
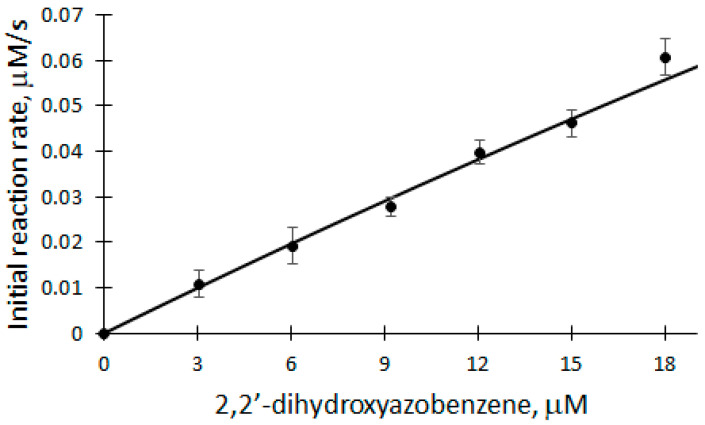
Dependence of the initial reaction rate of DHAB biocatalytic oxidation on DHAB concentration: 5 nM rCip, 100 μM H_2_O_2_, 3–18 μM DHAB.

The dependence of V_0_ on DHAB concentration was studied in the range of 3 to 18 μM at 25 °C in a 50 mM phosphate buffer solution of pH 7.2 (Figure 5).

A linear equation was used to analyze the DHAB dependence of V_0_ of the biocatalytic oxidation of DHAB (correlation coefficient r = 0.9968). The apparent bimolecular constant k_ox_ is calculated according to the formula:

(11) $$k_{\mathrm{ox}}=\frac{\mathrm{slope}\left([\mathrm{DHAB}],V_{0}\right)}{2{\left[E\right]}_{t}}$$

Applying Equation (11) and based on the parameters obtained from the dependence, the apparent bimolecular reactivity constant of DHAB with rCip equal to 3.182 × 10^5^ M^−1^s^−1^ was calculated. Also, the classical ping-pong peroxidase action scheme (Equations (1)–(3)) was used to analyze the dependence of the initial reaction rate of DHAB biocatalytic oxidation. Applying Equation (10), where k_1_ = 7.1 × 10^6^ M^−1^s^−1^ [32], the apparent bimolecular reactivity constant of DHAB with rCip was calculated to be 3.3 × 10^5^ M^−1^s^−1^. Also, after applying the model and calculating the theoretical V_max_, the turnover number of an enzyme (k_cat_) can be obtained, which in this case is equal to 660 s^−1^.

Summarizing the studies, it can be seen that very similar results have been obtained throughout. The apparent bimolecular constant calculated from direct experimental data is equal to (3.32 ± 0.14) × 10^5^ M^−1^s^−1^. The bimolecular constant of steady-state kinetics calculated by modeling the system where k_1_ = 7.1 × 10^6^ M^−1^s^−1^ [32] is equal to (3.31 ± 0.01) × 10^5^ M^−1^s^−1^. The apparent bimolecular constants, calculated from simulations and experiments, exhibit excellent agreement with each other. This alignment indicates that the model derived accurately represents real processes, and the obtained constants fully characterize these processes. 

The results obtained in this study on the oxidation of DHAB catalyzed by rCiP yield several notable benefits. Firstly, the observation that the initial reaction rates depend on the pH and pKa values of rCiP active forms adds an additional layer of information. This knowledge is valuable in predicting how environmental factors, such as variations in pH, might influence the efficacy of biocatalytic degradation processes over extended periods. Moreover, by establishing dependencies on enzyme, substrate and hydrogen peroxide concentrations, this study aids in quantifying and predicting the rates of azo dye degradation. This information is vital for assessing the long-term environmental impact of these compounds and designing strategies for their mitigation. Finally, the calculation of bimolecular constants for the enzyme’s interaction with DHAB provides a detailed understanding of the enzymatic process. This insight can be utilized to predict the enzyme’s behavior in complex environmental matrices, contributing to the assessment of long-term effects and potential risks associated with azo dye contamination. In summary, this study’s findings contribute to a more comprehensive understanding of the biocatalytic oxidation of DHAB, providing valuable data for assessing the long-term toxicity, carcinogenesis, and epigenetic effects of azo dyes in the environment. This information is crucial for developing strategies to mitigate the environmental impact of these compounds and safeguarding ecosystems and human health.

## 3. Materials and Methods

### 3.1. Materials

2,2′-dihydroxyazobenzene (Aldrich, Taufkirchen, Germany) was dissolved in ethanol (Stumbras, Kaunas, Lithuania). A phosphate buffer solution of sodium dihydrogen phosphate (Carl Roth, Karlsruhe, Germany) and disodium hydrogen phosphate (VWR Chemicals, Leuven, Belgium) was prepared using deionized water. The concentration of hydrogen peroxide was calculated from its absorbance at 240 nm using a molar absorption coefficient of 39 M^−1^cm^−1^ [33]. Recombinant *Coprinus cinereus* peroxidase (rCip) (Novozymes A/S, Copenhagen, Denmark) was used in the studies. The concentration of *Coprinus cinereus* peroxidase prepared in deionized water was determined from its absorbance at 405 nm using a molar absorbance of 109 mM^−1^cm^−1^ [32].

### 3.2. Methods

All materials were weighed using an ME 204 analytical balance (Mettler Toledo, Billerica, MA, USA). Spectrophotometric measurements were performed using a spectrophotometer (Genesys 10S UV-VIS, Menlo Park, CA, USA). Fluorimetric measurements were performed using a computerized Aminco Bowman luminescence spectrometer (Thermo Electron Corporation, Waltham, MA, USA). Variations in fluorescence intensity of 2,2′-dihydroxyazobenzene (DHAB) were measured at 423 nm excitation and 620 nm fluorescence wavelengths. Under these established conditions, the products of biocatalytic DHAB oxidation do not fluoresce. In order to investigate the dependence of the initial reaction rate of DHAB oxidation on pH, kinetic measurements were performed in 50 mM phosphate buffer solution (pH 5.8–8.0) in the presence of 12 μM DHAB, 100 μM H_2_O_2_ and 5 nM rCip at a temperature of 25 °C, in a thermostated (thermostatized for 5 min) 1 cm quartz glass cuvette. In order to study the dependences of the initial reaction rate of DHAB oxidation on the concentration of rCip, H_2_O_2_ and DHAB, kinetic measurements were performed in 50 mM phosphate buffer solution (pH 7.2) in the presence of 0–18 μM DHAB, 0–150 μM H_2_O_2_ and 0–6 nM rCip at 25 °C, thermostated (thermostatic 5 min) in a 1 cm quartz glass cuvette. The reaction was started by introducing the enzyme solution.

The resulting kinetic curves were normalized to the concentration of 2,2′-dihydroxyazobenzene used. The initial parts of the normalized kinetic curves (60 s) are approximated by applying a straight-line equation, where the tangent of the slope angle of the line (tg(α)) is the initial reaction rate. Computer program Mathcad 2001 Professional was employed for data processing. 

## Data Availability

All data are contained within the article.
